# Diagnosis and therapies for patients with cerebral palsy over the past 30 years: a bibliometric analysis

**DOI:** 10.3389/fneur.2024.1354311

**Published:** 2024-04-17

**Authors:** Lili Jiang, Weifang Yang, Huai Chen, Huangcheng Song, Song Zhang

**Affiliations:** ^1^Department of Outpatient, Hangzhou Children's Hospital, Hangzhou Normal University, Hangzhou, Zhejiang, China; ^2^Department of Radiology, Zhejiang Hospital of Integrated Traditional Chinese and Western Medicine, Hangzhou, Zhejiang, China; ^3^Department of Neurosurgery, Zhejiang Hospital of Integrated Traditional Chinese and Western Medicine, Hangzhou, Zhejiang, China; ^4^Department of Neurosurgery, People's Hospital of Haimen District, Nantong, Jiangsu, China; ^5^Department of Neurosurgery, Hangzhou Children's Hospital, Hangzhou Normal University, Hangzhou, Zhejiang, China

**Keywords:** cerebral palsy, bibliometric analysis, visual analysis, diagnosis, therapy

## Abstract

**Background:**

Currently, the incidence of cerebral palsy is high in newborns. However, the current methods for diagnosing and treating patients with cerebral palsy are complex and poorly targeted. Moreover, these studies lack the support of bibliometric analysis results.

**Objective:**

Our study focused on a bibliometric analysis of published papers on the diagnosis and treatment of patients with cerebral palsy. This study identified the primary authors, institutions, and countries involved in analyzing the status and trends of research on the diagnosis and treatment of patients with cerebral palsy. Additionally, the study also involved screening pathways related to cerebral palsy.

**Methods:**

The PubMed database was searched for publications on the diagnosis and treatment of patients with cerebral palsy between 1990 and 2023. R v4.2.2 and VOSviewer v1.6.18 software tools were utilized to perform bibliometric analysis and visualization.

**Results:**

There were 1,965 publications on cerebral palsy diagnosis and 5,418 articles on the qualified treatment strategies, and the annual number of publications also increased. The United States dominated in this field of research. Gregory Y.H. Lip and Patrizio Lancellotti published the most number of papers. The Cleveland Clinic published the most number of papers in the field. According to the analysis of the co-occurrence of keywords, we found that the main research directions were age, sex, disease diagnosis, and treatment. Newly emerging research has focused mainly on heart failure, which is related to valvular heart disease.

**Conclusion:**

The findings presented in this study offer valuable insights into ongoing research and potential future directions pertaining to cerebral palsy. These insights can assist researchers in identifying suitable collaborators and enhancing their investigations aimed at identifying the underlying molecular mechanisms associated with cerebral palsy, encompassing its etiology, preventive measures, and therapeutic interventions.

## Introduction

Cerebral palsy (CP) is not a clear, separate disease classification but rather an umbrella term that includes miscellaneous signs and symptoms that change with age. Cerebral palsy is diagnosed primarily through motor function and posture disorders and typically develops in early childhood and persists throughout life. These disorders are not progressive but change with age (1, 2). Motor dysfunction is a fundamental manifestation of cerebral palsy and frequently co-occurs with additional impairments encompassing sensory, perceptual, cognitive, communicative, and behavioral disorders; epilepsy; and secondary musculoskeletal complications (3). Cerebral palsy occurs in 2–3 infants out of 1,000 live births (4). There are multiple causes of brain damage that affect movement, posture, and balance. Motor disorders associated with cerebral palsy can be classified as spasms, motor disorders, ataxia, or mixed/other disorders (5). The symptoms of cerebral palsy include movement disorders, hip dislocations, balance difficulties, and hand dysfunction. In cases of cerebral palsy without a clear cause, magnetic resonance imaging may help diagnose brain damage when there is no clinical diagnosis. Once cerebral palsy is diagnosed, instruments such as the hair motor function classification system can be used to assess its severity and treatment response. The treatment of motor disorders associated with cerebral palsy includes intramuscular botulinum toxin A, medications, selective dorsal rhizotomy, and physiotherapy could be used to treat a wide range of muscle disorders. Patients with cerebral palsy often experience problems that are not related to movement and that need to be addressed in adulthood; these problems include cognitive impairment, seizures, pressure sores, osteoporosis, behavioral or emotional problems, and speech and hearing impairment (6).

The visual analysis software used for literature analysis included R v4.2.2 and VOSviewer, which play important roles in analyzing the current state of scientific research, detecting disciplinary frontiers, and selecting research directions. Bibliometrics can provide us with information on the most influential factors (including countries, institutions, and authors) in the field we want to study through relevant publications. In a manner, this approach could provide researchers with potentially favorable directions for their research. Currently, there is no research summarizing the treatment and diagnosis of cerebral palsy.

The identification of the relationships between the molecular and pathological levels of pathways and between the diagnosis and therapies used in patients with cerebral palsy can provide a basis for understanding the pathogenesis of cerebral palsy to some extent. However, there have been few bibliometric studies on the diagnosis/therapies of patients with cerebral palsy. Based on our bibliometric analysis, we generated data on cerebral palsy to provide insights for researchers who seek to discover new topics and directions.

## Materials and methods

### Data selection

We screened the PubMed database for the period from 1 January 1990 to 20 January 2023 to identify publications related to the diagnosis and treatment of patients with cerebral palsy. We considered only English-language publications. The title and abstract were reviewed and screened by two independent reviewers (Lili Jiang and Song Zhang), and disagreements were resolved.

### Data analysis and visualization

Key information (e.g., title, author, country/region, institution, keywords, and the year of publication) was derived from our included articles that met the inclusion criteria. The above variables were processed and visualized using Python v3.10.8, R v4.2.2, and VOSviewer v1.6.18.

VOSviewer was applied to perform the network analysis of the authors and frequent keywords. The minimum number of occurrences of a keyword was seven, which was the parameter of VOSviewer. A country collaboration map and various chart drawings based on the bibliometrix R package were constructed.

### Pathway enrichment analysis

We downloaded canonical pathway gene sets based on the Reactome pathway database from the GSEA database (7). The pathway enrichment analysis of the GSE183021 (8) dataset was performed using the *GSVA* R package (9). The differential gene expression analysis of pathway scores was performed based on the *limma* R package (10).

## Results

### General information

**Figure 1:**
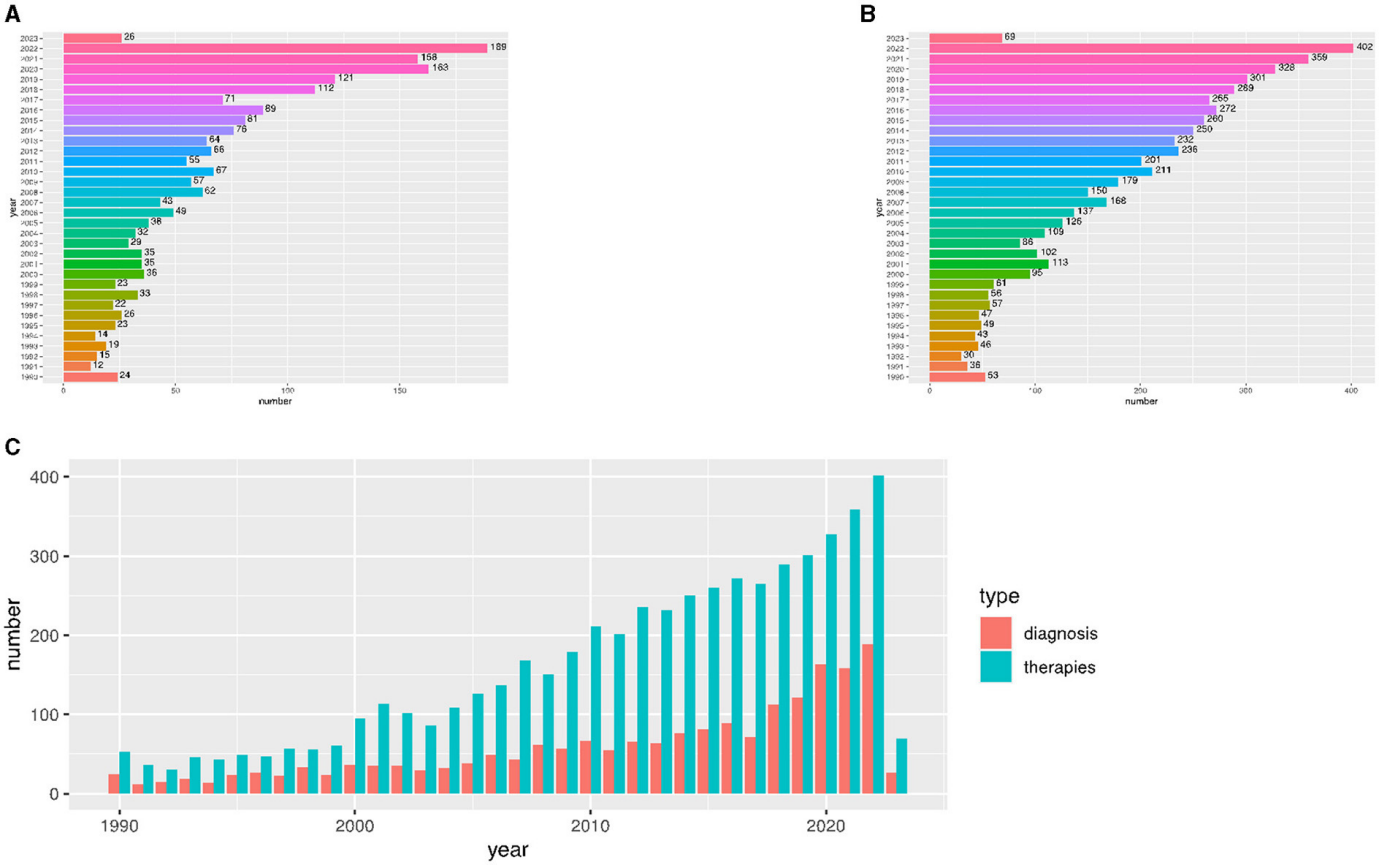
The number of annual publications relating to research on the diagnosis and treatment of cerebral palsy from 1990 to 2023. **(A)** The number of studies related to cerebral palsy diagnosis from 1990 to 2023. **(B)** The number of studies related to cerebral palsy treatment from 1990 to 2023. **(C)** A comparison of the annual journal publications related to cerebral palsy diagnosis and treatment.

A total of 7,383 publications were identified from PubMed. Of these, 1,965 publications were based on diagnosis, and 5,418 publications were based own treatment. We created two boxplots (Figure 1) to represent the number and ratio of annual publications over the last 33 years, which indicated the development trend of related research in this field. This field continues to attract the attention and interest of researchers, as evidenced by the growing number of publications. Research on the promotion of different diseases has shown differences in the associations between diagnosis and treatment.

Compared to diagnostic research, the treatment of cerebral palsy received more attention from 1990 to 2023. In summary, our results showed that the diagnosis and treatment of patients with cerebral palsy have gained widespread attention.

### Distribution of authors

**Figure 2:**
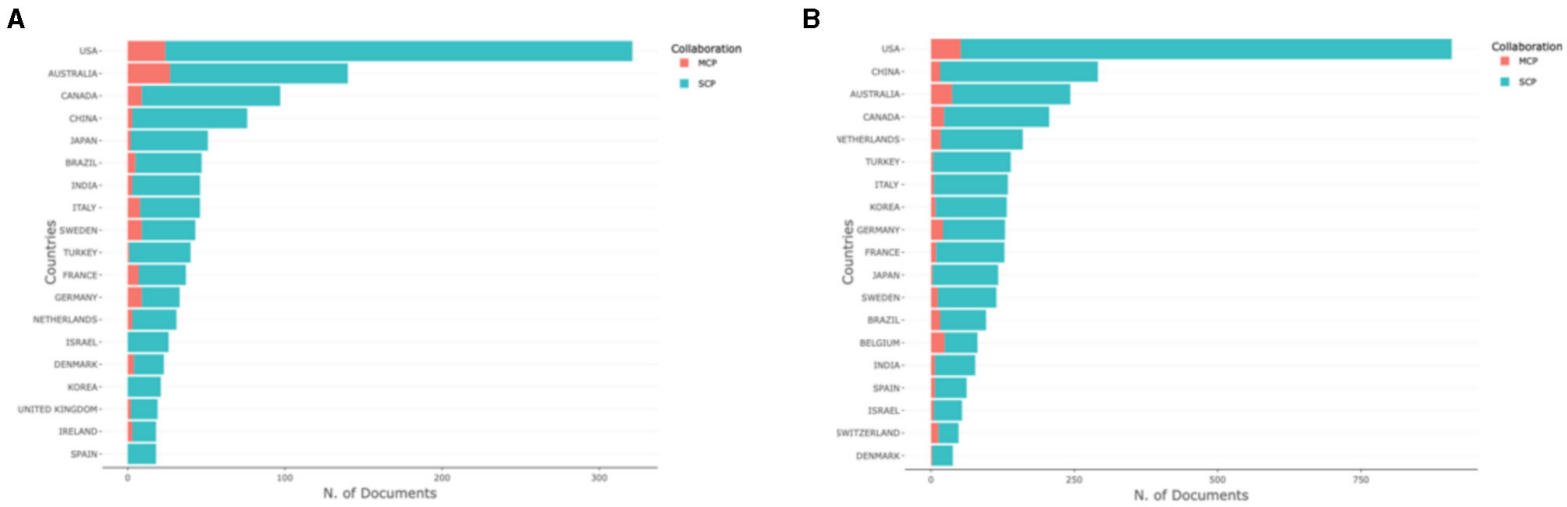
Boxplot of countries engaged in research on the diagnosis **(A)** and treatment **(B)** of cerebral palsy.

**Figure 3:**
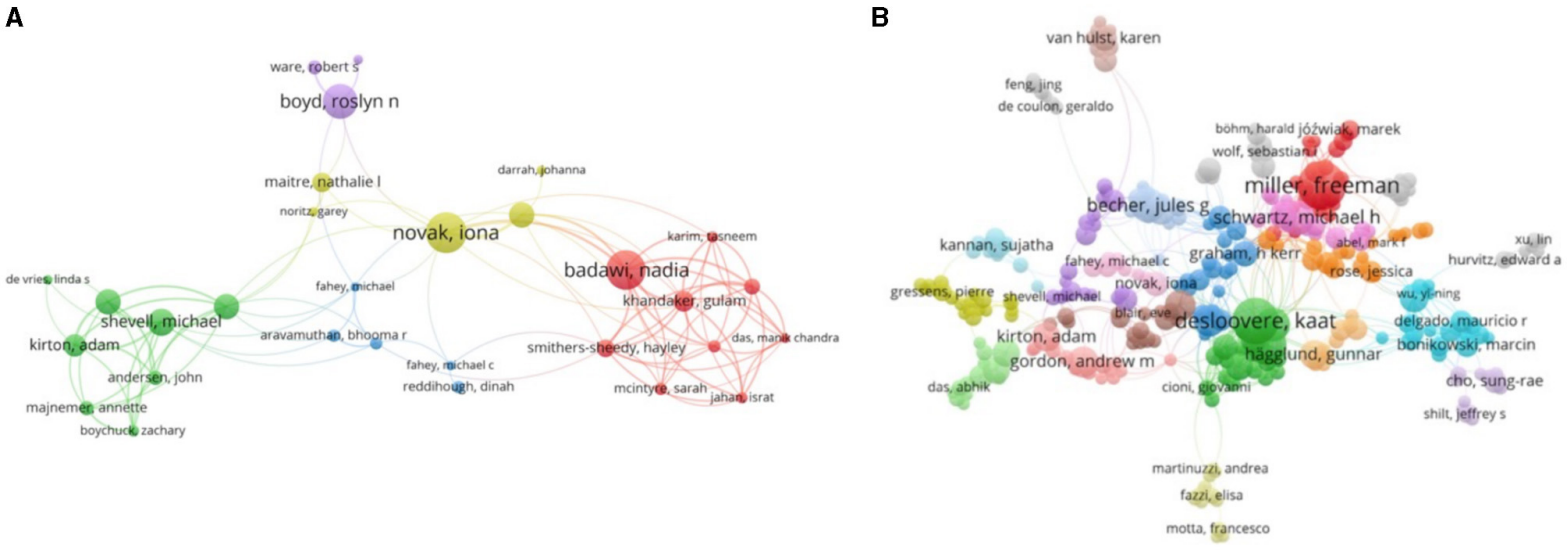
The network of authors who contributed to the research on the diagnosis **(A)** and treatment **(B)** of cerebral palsy. In the network, author contributions are reflected by node size. The connection strength is reflected by the thickness of the line.

The top 10 authors were involved in more than 10 papers on the diagnosis and treatment of patients with cerebral palsy, most of which were published in Europe and Australia (Tables 1, 2). The bar chart shows that most of the studies originated from the United States and were mainly single-country studies (Figure 2). The author collaboration network visualization (Figure 3) was performed on VOSviewer.

**Table 1:** The top 10 authors published articles on the diagnosis of cerebral palsy.

**Author**	**Documents**	**Total link strength**	**Country**
Novak, Iona	25	34	Australia
Basawi, Nadia	24	73	Britain
Boyd, Roslyn N	21	16	Australia
Shevell, Michael	16	44	Canada
Peterson, Mark D	16	28	The United States
Oskoui, Maryam	15	42	The United States
Fehlings, Darcy	15	38	Canada
Whitney, Daniel G	15	31	The United States
Morgan, Catherine	15	26	Australia
Kirton, Adam	13	38	The United States

**Table 2:** The top 10 authors published articles on the therapy for cerebral palsy.

**Author**	**Documents**	**Total link strength**	**Country**
Miller, Freeman	50	102	The United States
Desloovere, Kaat	47	184	Belgium
Molenaers, Guy	35	138	Belgium
Schwartz, Michael H	29	49	The United States
Becher, Jules G	28	69	The Netherlands
Van campenhout, Anja	27	122	Belgium
Boyd, Roslyn N	24	40	Australia
Elliott, Catherine	23	51	Britain
Gordon, Andrew M	23	29	The United States
Buiaer, Annemieke I	21	52	The Netherlands

### Distribution of countries/territories and institutions

**Figure 4:**
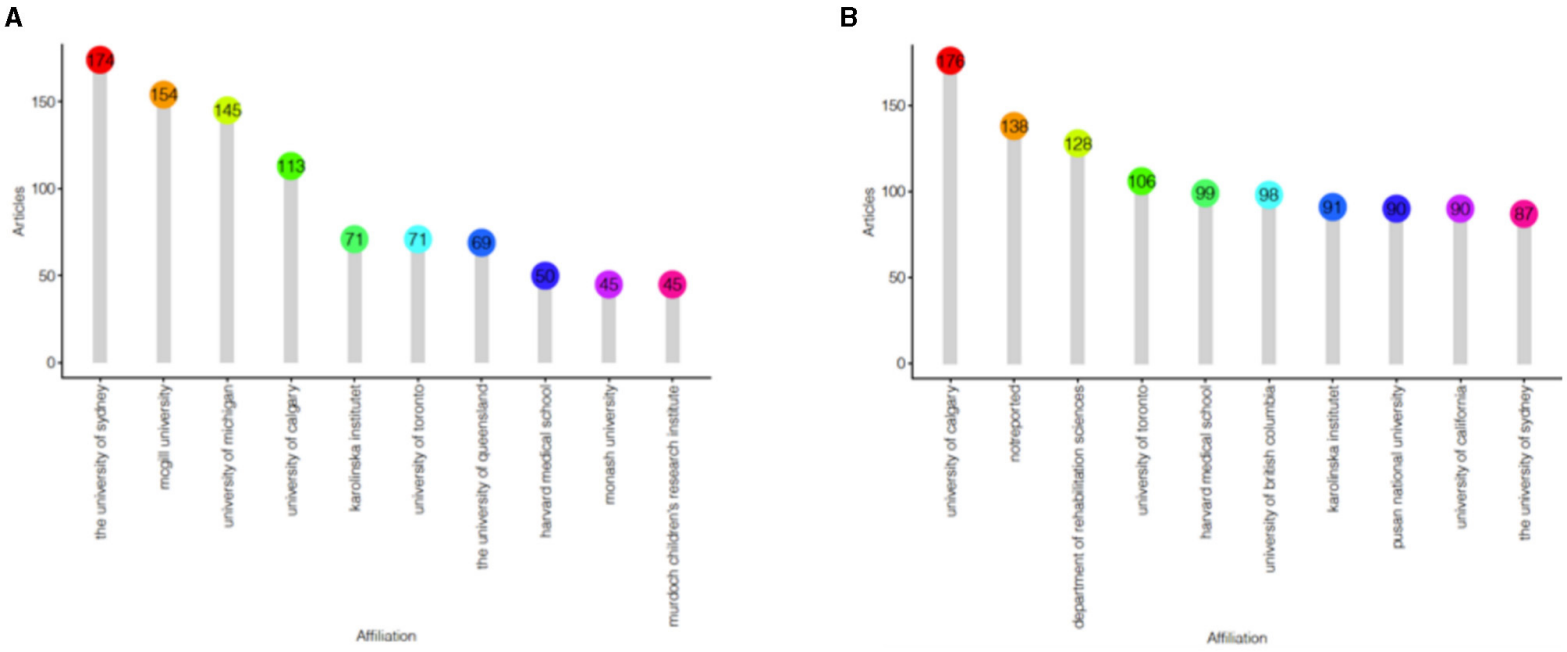
The treemap of institutions engaged in research on the diagnosis **(A)** and treatment **(B)** of cerebral palsy.

**Figure 5:**
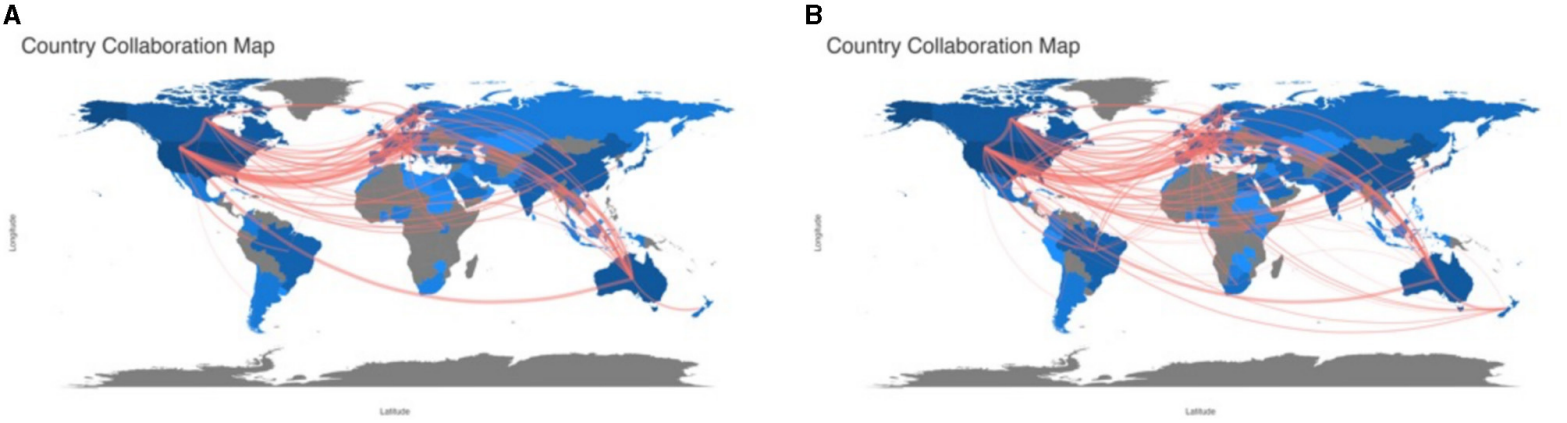
A country collaboration map of the research on the diagnosis **(A)** and treatment **(B)** of cerebral palsy.

The top 10 institutions had over 937 (Figure 4A) and 1,105 (Figure 4B) articles on the diagnosis and treatment of patients with cerebral palsy, respectively. According to the country collaboration map, we found that the research was mostly distributed in Europe and was closely related to many regions (Figure 5).

### Analysis of keywords and research direction analysis

**Figure 6:**
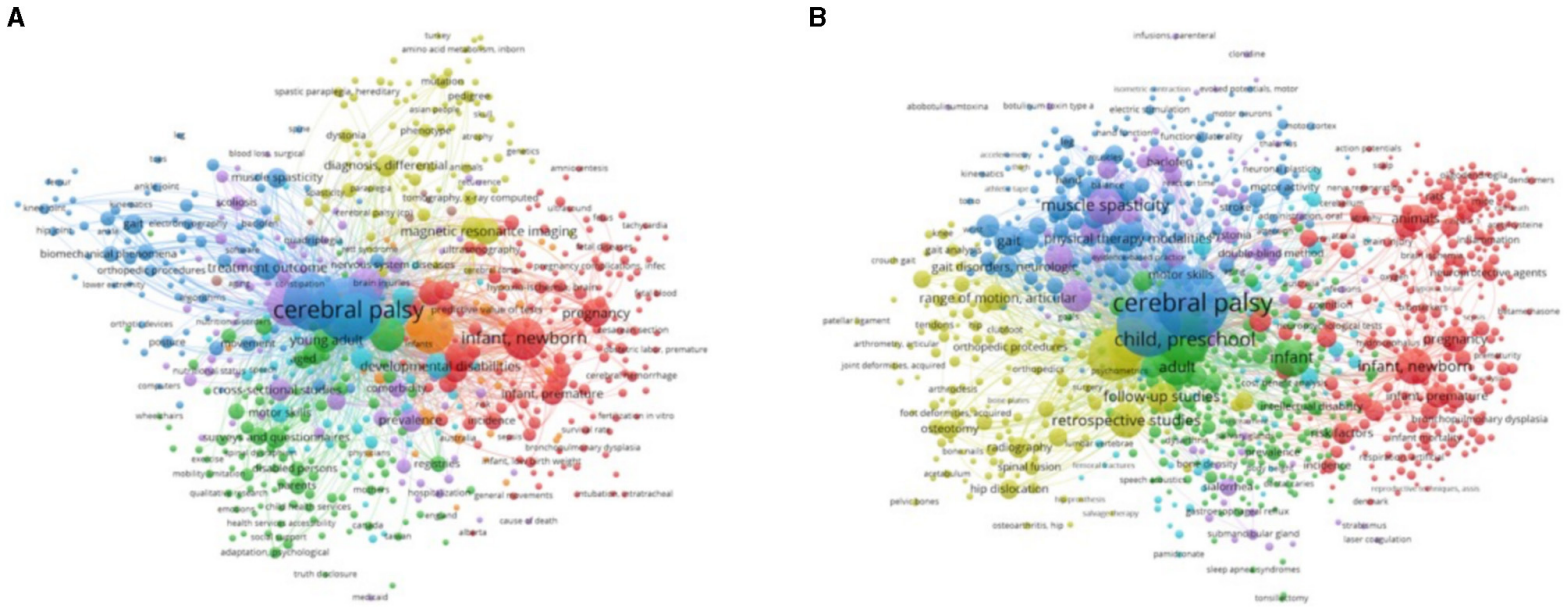
The network visualization of keywords related to the diagnosis **(A)** and treatment **(B)** of cerebral palsy. A keyword's weight is represented by the size of its circle. The distance between two circles indicates the relatedness between the two circles. Shorter distances are associated with stronger relatedness. The different colors of the circles represent the cluster classes.

The results of keyword analysis not only help us understand the topic of the article but also help us understand the hot spots in a research area. The top 20 keywords with the highest frequency are listed in Tables 3, 4. As shown in Figure 6, we used VOSviewer to display the network of keywords.

**Table 3:** The top 20 keywords for the diagnosis of cerebral palsy.

**Keyword**	**Occurrences**	**Total link strength**
Humans	1,654	16,072
Cerebral palsy	1,217	11,237
Female	1,047	11,723
Male	944	10,531
Child	884	9,154
Child, preschool	623	7,162
Infant	520	5,856
Adolescent	507	5,816
Infant, newborn	446	5,004
Retrospective studies	307	3,552
Adult	302	3,542
Risk factors	205	2,464
Pregnancy	177	2,023
Magnetic resonance imaging	152	1,549
Developmental disabilities	138	1,656
Quality Assessment of Diagnostic Accuracy Studies (QUADAS)	130	1,522
Treatment outcome	129	1,481
Young adult	127	1,682
Preferred Reporting Items for Systematic Reviews and Meta-analyses (PRISMA)	111	1,324
Neuroimaging	21	1,022

**Table 4:** The top 20 keywords related to therapy for cerebral palsy.

**Keyword**	**Occurrences**	**Total link strength**
Humans	4,474	46,659
Cerebral palsy	3,862	38,391
Child	2,806	31,157
Female	2,641	32,663
Male	2,483	30,801
Child, preschool	1,615	19,937
Adolescent	1,540	19,375
Treatment outcome	1,168	14,775
Infant	736	8,723
Muscle spasticity	697	7,917
Adult	692	8,546
Infant, newborn	667	7,460
Retrospective studies	592	7,682
Constraint-induced movement therapy (CIMT)	471	5,687
Follow-up studies	468	6,580
Rehabilitation very early in congenital hemiplegia (REACH)	413	4,867
Neuromuscular agents	371	4,572
Physical therapy modalities	335	3,774
Young adult	334	4,550
Animals	323	3,263

**Figure 7:**
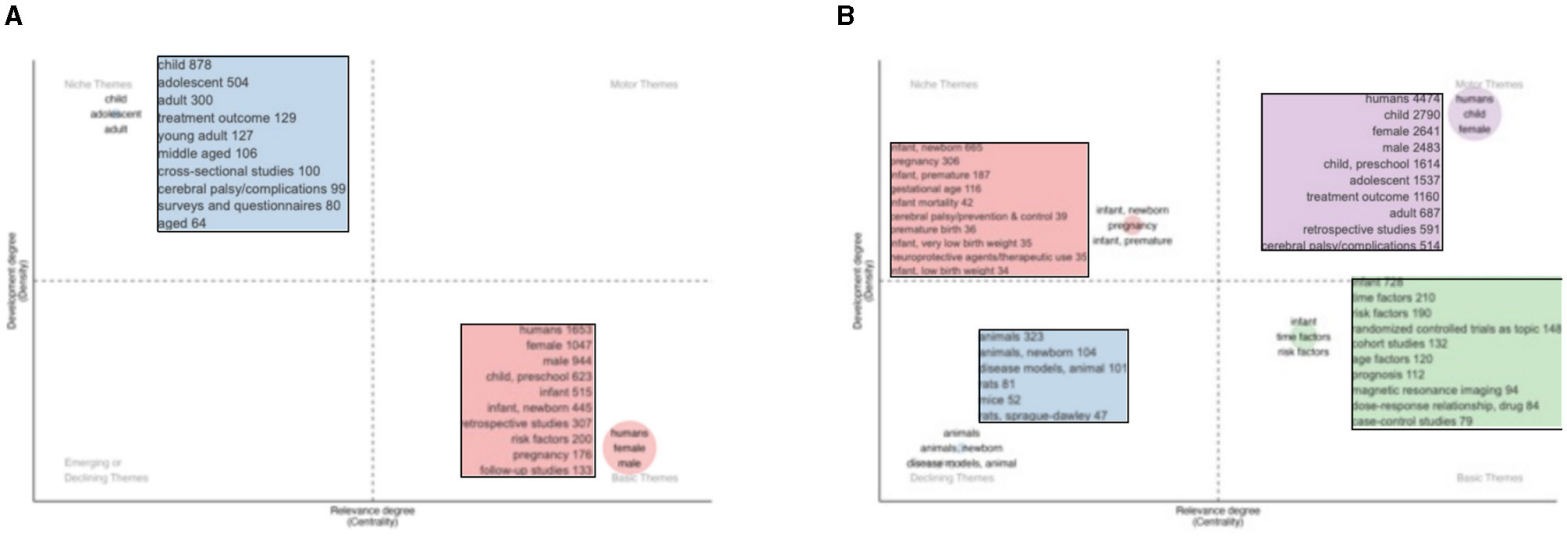
Thematic map of the research on the diagnosis **(A)** and treatment **(B)** of cerebral palsy. In the thematic map, the horizontal axis represents the centrality and the vertical axis represents the density. Motor themes: these themes are important and well-developed. Niche themes: these themes are very specialized/niche themes that are well-developed but are not important for the current field. Emerging or declining themes: these themes are not well-developed, may just emerge, or may soon disappear. Basic themes: these themes are very important to the field and have not achieved good development.

The heat map did not reveal relatively important results in the field of diagnosis, but, during treatment, we found that the individual may be more important in the clinical treatment of cerebral palsy (Figure 7).

### Pathway enrichment analysis

**Figure 8:**
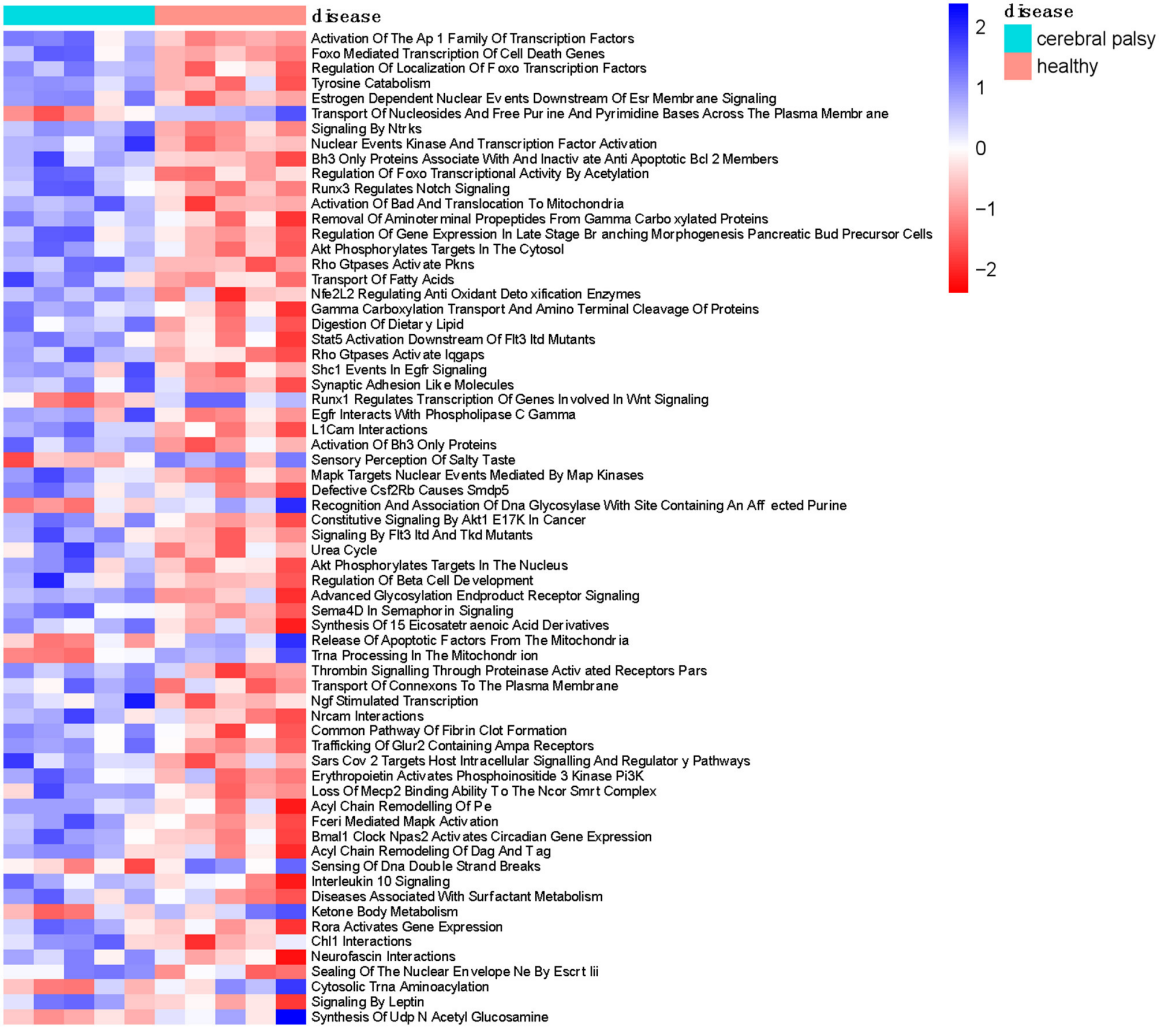
Pathway enrichment analysis of the expression matrix in cerebral palsy patients.

We downloaded the Reactome pathway dataset, subjected it to enrichment analysis to obtain the fractional matrix of the pathways, and conducted differential analysis to obtain the differential heatmap of cerebral palsy-related pathways (Figure 8).

We identified 3,089 pathways enriched in cerebral palsy, including 56 upregulated pathways (logFC > 0, *P* < 0.01) and 10 downregulated pathways (logFC < 0, *P* < 0.01).

## Discussion

In relation to publishing patterns, our analysis reveals a steady upward trajectory in the number of publications pertaining to this domain since 1990. This trend suggests that both fields will continue to garner significant interest from researchers in the coming years. Moreover, the authors' collaborative network analysis offers a valuable tool for researchers to foster collaborative associations, as it enables the identification of influential research groups and potential partners.

Most of the authors listed in Tables 1, 2 are located in Europe/Australia, and the relationships between European scholars are much closer than those between scholars in other regions. As shown in Figure 2, many authors have made contributions to this field, but their cooperation needs to be strengthened. Figure 4 shows that most well-published institutions are located in the United States. For the current research, the active cooperation and exchange of ideas between researchers/research institutions in various countries will be beneficial to the development of this field.

### Study hotspots

We have not mined important research directions from the diagnosis section, but we have found that the basic themes for the diagnosis section are similar to the motor themes for the treatment section. The basic criteria for diagnoses included studies on humans (1,653), female (1,047), male (944), child, preschool (623), infant (515), infant or newborn (445) factors; retrospective studies (307); information on risk factor (200); pregnancy (176); and follow-up studies (133).

The motor themes for the treatment included studies on humans (4,474), child (2,790), female (2,641), male (2,483), child, preschools (1,614), adolescent (1,537), treatment outcome (1,160), adult (687), retrospective studies (591), and cerebral palsy/complications (514). However, to some extent, these results do not explain some of the details, thus we combined them with keyword analysis.

Research has shown that cerebral palsy or “high risk of cerebral palsy” can be accurately predicted before the corrected age of 6 months, and detecting cerebral palsy before the corrected age, including magnetic resonance imaging (MRI), has the best predictive effectiveness, with a sensitivity of 86–89% (11).

Magnetic resonance imaging (MRI) can help assess the duration of brain damage during the development of cerebral palsy (CP) (12). MRI can also be used to assess white matter damage in premature infants and is therefore more common in the field of cerebral palsy diagnosis (13).

Therefore, the development of MRI technology and the discovery of cerebral palsy monitoring indicators have become increasingly important. In the biological and molecular research fields, finding more accurate monitoring indicators is particularly important (14, 15).

The keywords used for the treatment of cerebral palsy include muscle spasticity and physical therapy modifications. The treatment of cerebral palsy (CP) includes physical therapy and complementary therapies to various standard clinical treatments (16).

The prenatal diagnosis of cerebral palsy relies on routine ultrasound examinations, which mainly detect congenital malformations and other diseases, but there is currently no specific method for treating cerebral palsy (17). Research (18) has summarized the risk factors for cerebral palsy, such as chorioamnionitis, maternal infection, neurotropic virus infection, any virus from Herpes Group B for hemiplegia, and infection of the mother with cytomegalovirus during the first trimester.

Therefore, in the absence of significant progress in imaging, biochemistry, and other related research fields, the discovery of key genes and therapeutic targets in genetics for cerebral palsy will promote the development of methods for diagnosing and treating cerebral palsy.

### Future frontiers

Due to the extensive screening of diagnosis and treatment, we observed a relatively significant clinical situation. Imaging is used for the diagnosis of cerebral palsy, while physical therapy and some auxiliary treatments are used for the clinical treatment of cerebral palsy. At the molecular level, research on cerebral palsy

is relatively scarce. Currently, relatively few studies on neurons, muscle contractures, and genes exist; these studies have not been fully examined through bibliometric analysis of the literature. Research on the underlying mechanism of cerebral palsy is not particularly abundant, but, in combination with other research studies on diseases and a small amount of basic research, we speculate that research on the molecular mechanisms will provide evidence for the diagnosis and treatment of cerebral palsy to some extent.

The “Activation of the Ap 1 Family of Transcription Factors” was the most significantly differentially expressed pathway. A previous study showed that gene mutations in AP-1-related transcription factor complexes can affect brain development (19).

The “Akt Phosphorylates Targets in the Cytosol” represents the presence of phosphorylated Akt in the cytoplasm. Studies have shown that the phosphoinositide 3-kinase (PI3K)-protein kinase B (Akt) signaling pathway enhances neurogenesis (20). Our study showed that Akt phosphorylation was downregulated in patients with NAFLD compared to healthy individuals (Figure 8).

## Conclusion

This study examined a comprehensive collection of 7,383 publications spanning from 1990 to 2023 that specifically address valvular diagnosis and therapies in patients with cerebral palsy. The objective of this research is to identify the countries, institutions, and authors that have had significant impacts on this field. Furthermore, our investigation focuses on specific topics to discern prevailing research patterns. Additionally, we employed GSVA R package to explore common pathways associated with cerebral palsy. Although cerebral palsy is a congenital disease, clinical detection has been less common but should be the focus of future research. Although the molecular mechanisms involved have not been the focus of current research, these mechanisms will provide a basis for the drug treatment to some extent.

## Data availability statement

The original contributions presented in the study are included in the article/Supplementary material, further inquiries can be directed to the corresponding author.

## Author contributions

LJ: Project administration, Writing – original draft. WY: Writing – original draft. HC: Data curation, Writing – review & editing. HS: Data curation, Writing – review & editing. SZ: Conceptualization, Data curation, Formal analysis, Funding acquisition, Investigation, Methodology, Project administration, Resources, Software, Supervision, Validation, Visualization, Writing – original draft, Writing – review & editing.

## Supplementary material

Figure S1 A flowchart of literature identification and selection.
