# Supplementary figures and images for: Diagnosis and therapies for patients with cerebral palsy over the past 30 years: a bibliometric analysis
Source: Front Neurol. 2024 Apr 17;15:1354311. doi: 10.3389/fneur.2024.1354311 (PMC11061478; doi:10.3389/fneur.2024.1354311)

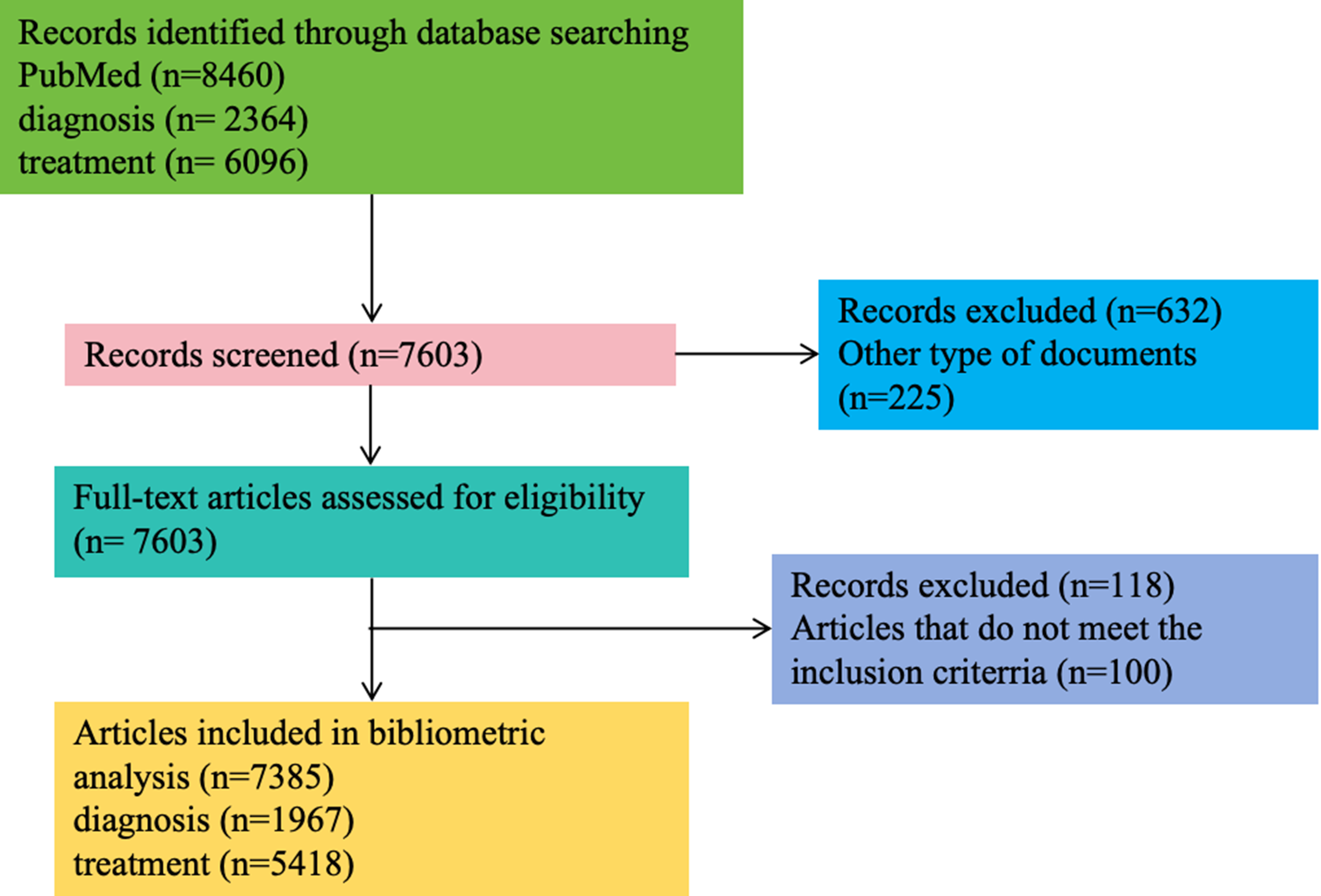

Supplement: Figure S1 — A flowchart of literature identification and selection. [file Image_1.tif]
